# Monitoring Recently Acquired HIV Infections in Amsterdam, The Netherlands: The Attribution of Test Locations

**DOI:** 10.3389/frph.2021.568611

**Published:** 2021-02-10

**Authors:** Isabel A. L. Slurink, Frank van de Baan, Ard I. van Sighem, Alje P. van Dam, Thijs J. W. van de Laar, Godelieve J. de Bree, Birgit H. B. van Benthem, Eline L. M. Op de Coul

**Affiliations:** ^1^National Institute for Public Health and the Environment, Centre for Infectious Disease Control, Epidemiology and Surveillance, Bilthoven, Netherlands; ^2^Stichting HIV Monitoring, Amsterdam, Netherlands; ^3^Public Health Service Amsterdam, Netherlands; ^4^OLVG Hospital, Amsterdam, Netherlands; ^5^Department of Donor Medicine Research, Laboratory of Blood Borne Infections, Sanquin Research, Amsterdam, Netherlands; ^6^Department of Internal Medicine, Amsterdam University Medical Centers, Location AMC, Amsterdam, Netherlands

**Keywords:** HIV, recent infection, MSM, The Netherlands, avidity assay

## Abstract

**Background:** Surveillance of recent HIV infections (RHI) using an avidity assay has been implemented at Dutch sexual health centres (SHC) since 2014, but data on RHI diagnosed at other test locations is lacking.

**Setting:** Implementation of the avidity assay in HIV treatment clinics for the purpose of studying RHI among HIV patients tested at different test locations.

**Methods:** We retrospectively tested leftover specimens from newly diagnosed HIV patients in care in 2013–2015 in Amsterdam. Avidity Index (AI) values ≤0.80 indicated recent infection (acquired ≤6 months prior to diagnosis), and AI > 0.80 indicated established infection (acquired >6 months prior to diagnosis). An algorithm for RHI was applied to correct for false recency. Recency based on this algorithm was compared with recency based on epidemiological data only. Multivariable logistic regression analysis was used to identify factors associated with RHI among men who have sex with men (MSM).

**Results:** We tested 447 specimens with avidity; 72% from MSM. Proportions of RHI were 20% among MSM and 10% among heterosexuals. SHC showed highest proportions of RHI (27%), followed by GPs (15%), hospitals (5%), and other/unknown locations (11%) (*p* < 0.001). Test location was the only factor associated with RHI among MSM. A higher proportion of RHI was found based on epidemiological data compared to avidity testing combined with the RHI algorithm.

**Conclusion:** SHC identify more RHI infections compared to other test locations, as they serve high-risk populations and offer frequent HIV testing. Using avidity-testing for surveillance purposes may help targeting prevention programs, but the assay lacks robustness and its added value may decline with improved, repeat HIV testing and data collection.

## Introduction

In January 2014, the National Institute for Public Health and the Environment (RIVM) implemented a biomarker-assay (Architect avidity) to distinguish recently acquired HIV infections (≤6 months prior to diagnosis) from established HIV infections (acquired >6 months prior to diagnosis) in routine HIV surveillance at sexual health centers (SHCs) in the Netherlands (1, 2). The goal of this enhanced surveillance is to monitor trends in recent HIV infections (RHI) for planning and evaluating local and national HIV prevention programs. RHI, especially those in the acute phase, are associated with higher viral loads and contribute disproportionately to HIV transmission (3, 4). Therefore, delayed diagnosis and treatment of individuals with RHI may have significant individual and public health implications (5).

The Architect avidity assay is one of several assays that have been developed to distinguish recently acquired from established HIV infections (6, 7). Following the recommendations from the European Center for Disease Prevention and Control (ECDC), various European countries implemented a biomarker-assay of which the most commonly used are the Less-sensitive/Detuned Vitros, the BED CEIA (a capture enzyme immunoassay), the avidity assay (Lag-Avidity EIA, Architect HIV ag/ab combo or Bio-Rad avidity), and the INNO-LIA (p31 antigen immunoblot)) (8–13). These assays can be combined with clinical data resulting in a testing algorithm (RITA: recent infection testing algorithm). Differences in the performance of the assays vary due to differences in the recency windows of the assays (13). Furthermore, previous studies showed that avidity assays are less affected by HIV-1 subtype variation when compared to the BED immune assays, supporting the use of avidity assays for RHI monitoring. Results from an independent evaluation of the performance of five individual HIV incidence assays showed that the Lag-Avidity assay had the lowest false recency rate (14). The first results from the Dutch enhanced surveillance at SHCs showed high proportions of RHI (30–40%) among men who have sex with men (MSM) and proportions of 10–11% for heterosexuals (1, 15). However, of all individuals entering HIV care in the Netherlands, only 30% are diagnosed at SHC (16). Therefore, a better understanding is needed on the number of RHI among individuals diagnosed at other test locations, such as general practices (GPs) and hospitals where 28%, respectively 27% of all new HIV cases are diagnosed. We conducted a pilot study in the Amsterdam region to assess the feasibility of implementing the avidity assay for RHI at HIV treatment centers, where all newly diagnosed HIV patients from various test locations in this region are registered and treated. For this region, where 22% of all new HIV infections in the Netherlands are diagnosed (16), we collected leftover specimens from newly diagnosed patients in care in 7 treatment centers. The samples were tested with the avidity assay and subsequently corrected for false recency with the RITA algorithm of the ECDC, that includes the clinical data of AIDS-defining illness, CD4 count, and viral load (17).

Last, we compared the performance of this algorithm for biomarker-assays with routinely collected epidemiological data only that can be used as indicators for recent infection, such as (1) the date of the last negative HIV test in the past 6 months, (2) CD4 of ≥500 cells/mm^3^ at diagnosis as described by Le Guillou et al. (18) reclassified as established infection in presence of AIDS-defining illnesses or viral load (<400 copies/ml), and (3) a combination of these two epidemiological indicators, to assess the added value of the HIV avidity assay for surveillance purposes in an era of improved HIV testing and more comprehensive data collection.

## Methods

### Specimen and Data Collection

In 2016–2017, residual plasma or serum specimens from individuals newly diagnosed with HIV-1 between 2013 and 2015 from 7 HIV treatment centers in Amsterdam, were retrospectively collected and tested with an avidity assay (7, 19). Eligibility criteria for inclusion were: >16 years of age at diagnosis, and having a stored serum or plasma sample (volume >200 μl) within 4 weeks after HIV diagnosis and before ART treatment. Demographic and clinical data were retrieved from the national ATHENA database maintained by Stichting HIV Monitoring (SHM). The AIDS Therapy Evaluation in the Netherlands (ATHENA) national observational HIV cohort (20) was initiated in 1998 and captures clinical data from >98% of all patients with diagnosed HIV infection who are in HIV care in the Netherlands. The data collection is continuous, and the database of the ATHENA cohort is updated twice a year. ATHENA is an open cohort, as new participants continue to be enrolled on entry into HIV care, following a positive HIV diagnosis. For our study data are extracted from medical records from the patients registered in Amsterdam. Variables included were age, gender, mode of transmission, region of origin (Western or non-Western), location of HIV diagnosis or referral (SHC, GP, hospital or other/unknown location), presumed country of infection, area of residence (Amsterdam or other/unknown) at the time of entering HIV care, (self-reported) date of last HIV negative test, CD4 count at diagnosis, viral load at diagnosis, and presence of AIDS-defining illness at diagnosis (16). The classification of “Western/non-Western” refers to the geographic region of origin based on the patients' country of birth. The “Western” region includes western and central Europe, North America, and the Australian/Pacific region. All other regions were classified as “non-Western.”

### Laboratory Procedures

Avidity testing was performed using a standardized operation procedure (SOP) in a routine diagnostic laboratory. Procedures for avidity testing have been described elsewhere (7, 19). In short, specimens were tested with an anti-HIV avidity assay using a 4th generation commercial enzyme immunoassay (EIA), the Architect HIV Ag/Ab Combo (Abbott Diagnostics, Wiesbaden, Germany). Cut-off values of the Avidity Index (AI) were: ≤0.80 for recent infection (acquired ≤6 months prior to diagnosis), and AI > 0.80 for established infection (acquired >6 months prior to diagnosis). Each run included two quality controls (QCs): a negative control (NC) and a (weakly) positive control (PC) with an AI just below the cut-off of 0.80. In case of either a positive NC or a negative/gray-zone PC test results were rejected and the whole run was repeated.

The ECDC RITA algorithm for newly diagnosed patients was applied to minimize misclassification of established infections as recent, e.g., reduce the false recent rate (FRR) in subsequent steps: (1) reclassify RHI as established infection if the patient has an AIDS-defining illness, (2) RHI reclassified as established infection as CD4 count <200 cells/mm^3^, and (3) RHI reclassified as established infection as viral load <400 copies/ml at HIV diagnosis (17).

### Statistical Methods

Chi-square tests were used to compare characteristics between individuals with and without a RITA result (mostly due to lack of available sample), and between individuals with a recent and established HIV infection. Linear regression analysis was performed to assess trends in proportions of RHI from 2013 to 2015. Univariable and multivariable logistic regression analyses were used to identify factors associated with RHI for MSM only, due to low numbers of RHI among heterosexuals. Variables included in the univariable model were year of diagnosis, region of origin, country of infection, area of residency, and test location. The multivariable final model was obtained from backward stepwise deletion based on the probability of likelihood-ratio statistics using conditional parameter estimates. *P*-values under 0.05 were considered statistically significant.

Lastly, proportions of RHI after applying the RITA algorithm were compared with proportions of RHI defined as (1) prior negative HIV test result ≤6 months before diagnosis, (2) CD4 count ≥500 cells/mm^3^ (18), reclassified as established infection in presence of AIDS-defining illnesses or viral load (<400 copies/ml) at HIV diagnosis, and (3) 1 & 2 combined, i.e., option 2 corrected for false established cases based on last negative test result ≤6 months before diagnosis. Data were analyzed using IBM-SPSS 21 (IBM SPSS Statistics for Windows, version 24, IBM Corp., Armonk, N.Y., USA).

## Results

### New HIV Diagnoses and Avidity Testing

**Figure 1:**
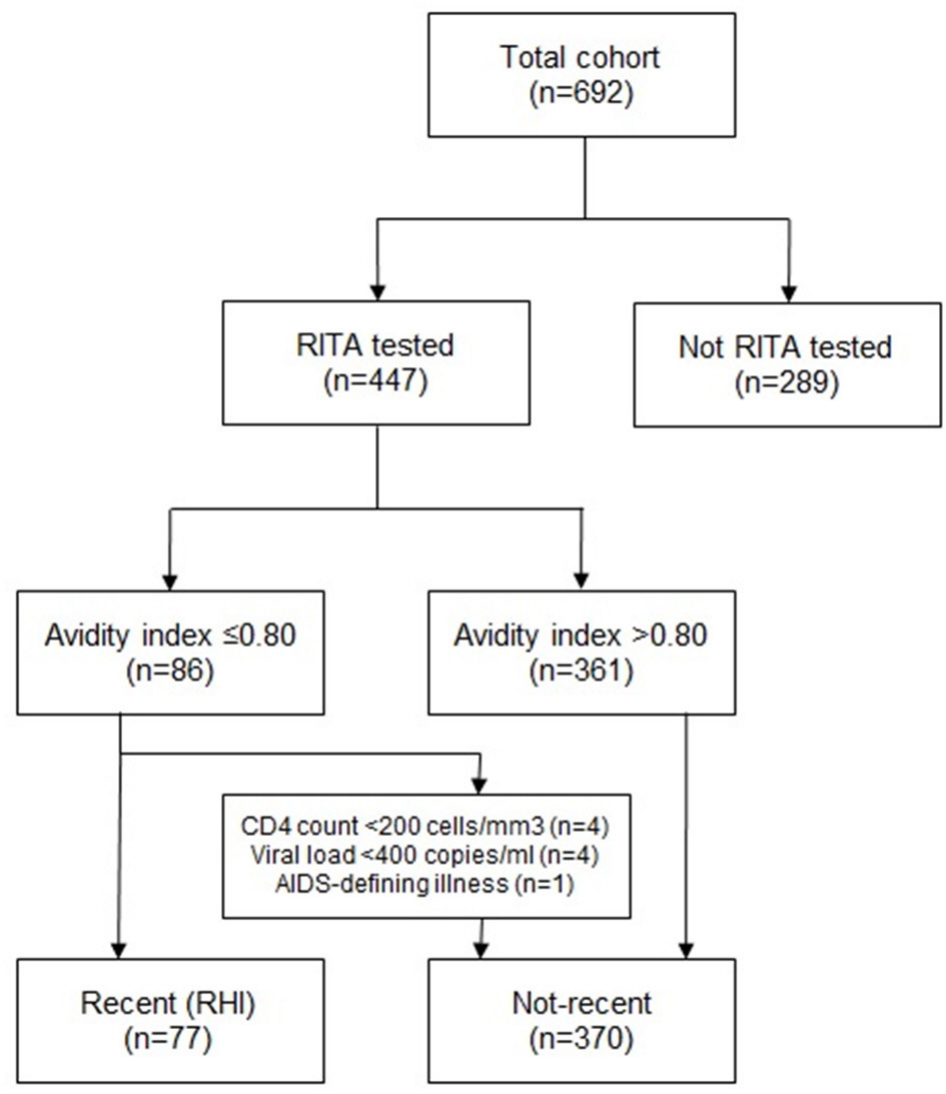
Flowchart on HIV-patients included in the study, samples collected, and samples tested with the Avidity Assay and numbers of recent HIV-infections identified.

Between 2013 and 2015, 692 newly diagnosed HIV patients in the Amsterdam region were registered in the ATHENA database, of whom the median age was 40 years (range 18–77 years) (Table 1). MSM accounted for 73.6% of diagnoses, heterosexual males for 11.6% and heterosexual females for 10.5%. The majority of HIV patients was diagnosed at a SHC (39.0%), followed by GPs (27.5%) and hospitals (26.6%). A specimen for avidity testing was available for 447 patients (64.6%) (Figure 1).

**Table 1:** Characteristics of newly diagnosed HIV patients according to being RITA tested and to RITA result, region of Amsterdam, 2013–2015.

		**Total cohort**			**RITA result**		
	**Total cohort (*n* = 692) (%)**	**RITA tested (*n* = 447) (%)**	**Not RITA tested (*n* = 289) (%)**	***p*-value**	**Recent (*n* = 77) (%)**	**Non-recent (*n* = 370) (%)**	***p*-value**
Year of diagnosis							
2013	213 (30.8)	167 (37.4)	62 (21.5)	<0.001	31 (40.3)	136 (36.8)	0.24
2014	232 (33.5)	158 (35.3)	84 (29.1)		21 (27.3)	137 (37.0)	
2015	247 (35.7)	122 (27.3)	143 (49.5)		25 (32.5)	97 (26.2)	
Age (years)							
Median (IQR)	39 (18)	40 (17)	37 (20)	0.21	34 (58)	41 (53)	0.94
Age group							
≤ 24	67 (9.7)	41 (9.2)	26 (10.6)	0.65	11 (14.3)	30 (8.1)	0.02
25–34	197 (28.5)	125 (28.0)	72 (29.4)		30 (39.0)	95 (25.7)	
35–44	180 (26.0)	123 (27.5)	57 (23.3)		17 (22.1)	106 (28.6)	
≥45	248 (35.8)	158 (35.3)	90 (36.7)		19 (24.7)	139 (37.6)	
Mode of transmission							
MSM	509 (73.6)	323 (72.3)	186 (75.9)	0.77	66 (85.7)	257 (69.5)	0.03
Heterosexual male	80 (11.6)	55 (12.3)	25 (10.2)		5 (6.5)	50 (13.5)	
Heterosexual female	73 (10.5)	49 (11.0)	24 (9.8)		5 (6.5)	44 (11.9)	
Unknown	30 (4.3)	20 (4.5)	10 (4.1)		1 (1.3)	19 (5.1)	
Region of origin							
Western	433 (62.6)	263 (58.8)	170 (69.4)	0.007	50 (64.9)	213 (57.6)	0.39
Non-Western	252 (36.4)	181 (40.5)	71 (29.0)		27 (35.1)	154 (41.6)	
Unknown	7 (1.0)	3 (0.7)	4 (1.6)		0 (0.0)	3 (0.8)	
Country of infection							
Netherlands	352 (50.9)	231 (51.7)	121 (49.4)	0.56	48 (62.3)	183 (49.5)	0.04
Other/unknown	340 (49.1)	216 (48.3)	124 (50.6)		29 (37.7)	187 (50.5)	
Area of residence							
Amsterdam	531 (76.7)	338 (75.6)	193 (78.8)	0.35	61 (79.2)	277 (74.9)	0.42
Other/unknown	161 (23.3)	109 (24.4)	52 (21.2)		16 (20.8)	93 (25.1)	
Test location							
Sexual health center	270 (39.0)	175 (39.1)	95 (38.8)	0.39	48 (62.3)	127 (34.3)	<0.001
General practice	184 (26.6)	115 (25.7)	69 (28.2)		20 (26.0)	110 (29.7)	
Hospital	190 (27.5)	130 (29.1)	60 (24.5)		6 (7.8)	109 (29.5)	
Other/unknown	48 (6.9)	27 (6.0)	21 (8.6)		3 (3.9)	24 (6.5)	

Characteristics of patients with and without an avidity test result were similar regarding age, mode of transmission, country of infection, area of residency, and test location. However, patients with an avidity test result more often originated from a non-Western region (40.5% vs. Western 29.0%, *p* < 0.01) compared to patients without an avidity test result. Furthermore, the proportion of patients with an avidity test result decreased over time from 78 (2013), 68 (2014) to 49% (2015) (*p* < 0.001).

### Proportions of Recent Infections

Characteristics according to proportions of RHI among MSM and heterosexuals are displayed in Table 2. Of the 447 patients with an avidity result, 86 (19.2%) patients had an avidity index (AI) of ≤0.80; an indication for RHI. Data on CD4 count and HIV viral load were available for 97.3 and 96.9% of patients with an avidity test result. Applying the RITA algorithm, nine patients were reclassified from recent to established HIV-infection based on AIDS-defining illness (*n* = 1), CD4 count (*n* = 4), or viral load (*n* = 4). This resulted in 77 patients (17.2%) classified as having a RHI, with a false recent rate of 10.5% (Figure 1).

**Table 2:** Proportions of recent HIV infections among heterosexuals and MSM, and associations of recent HIV infections among MSM by univariable and multivariable logistic regression analysis, region of Amsterdam, 2013–2015.

	**Heterosexuals**	**MSM**				
	**Recent infection**	**Recent infection**	**Univariate analysis**		**Multivariate analysis**	
	**% (n/N)**	**% (n/N)**	**OR (95%CI)**	***p*-value**	**OR (95%CI)**	***p*-value**
**Year of diagnosis**						
2013	11.4 (4/35)	21.3 (27/127)	REF			
2014	2.9 (1/34)	16.9 (20/118)	0.76 (0.40; 1.44)	0.39		
2015	14.3 (5/35)	24.4 (19/78)	1.2 (0.61; 2.3)	0.61		
**Age group**						
≤ 24	28.6 (2/7)	26.5 (9/34)	REF			
25–34	11.1 (3/27)	28.0 (26/93)	1.08 (0.44; 2.62)	0.87		
35–44	6.7 (2/30)	17.0 (15/88)	0.57 (0.22; 1.47)	0.24		
≥45	7.5 (3/40)	14.8 (16/108)	0.48 (0.19; 1.22)	0.12		
**Region of origin** ^**1**^						
Western	7.7 (3/39)	21.8 (46/221)	REF			
Non-Western	10.9 (7/64)	18.2 (20/110)	0.80 (0.44; 1.43)	0.45		
**Country of infection**						
Netherlands	12.8 (5/34)	23.6 (42/178)	REF			
Other/unknown	7.7 (5/60)	16.8 (24/143)	0.652 (0.37; 1.14)	0.13		
**Area of residency**						
Amsterdam	11.1 (8/72)	20.2 (52/257)	REF			
Other/unknown	6.3 (2/32)	21.9 (14/64)	1.15 (0.57; 2.17)	0.75		
**Test location**						
Sexual health center	8.3 (1/12)	28.9 (46/159)	REF		REF	
General practice	16.2 (6/37)	15.4 (14/91)	0.45 (0.23; 0.88)	0.02	0.45 (0.23; 0.88)	0.02
Hospital	4.9 (2/41)	6.8 (4/59)	0.18 (0.06; 0.52)	0.002	0.18 (0.06; 0.51)	0.002
Other/unknown	7.1 (1/14)	16.7 (2/12)	0.50 (0.11; 2.35)	0.38	0.50 (0.11; 2.35)	0.37

1 *One heterosexual and two MSM with unknown region of origin, all non-recent HIV infections*.

*Missing values (Other/unknown) were included as category in the regression analysis in case of >5%*.

Patients with RHI, as defined by the RITA algorithm, were younger, more often MSM, more likely to have acquired their infection in the Netherlands, and were more often diagnosed at a SHC compared to patients with an established HIV infection (Table 1). Overall, proportions of RHI did not change considerably over the study years (Supplementary Table 1). Proportions of RHI at SHCs tended to be higher in 2015 (42.1%) compared to 2013–2014 (24.2–22.5%, *p* = 0.09). Proportions of RHI diagnosed at GPs fluctuated over the years (22.2, 6.7, 16.1%), but numbers were small.

The proportion of MSM with a RHI was 20.4% (66/323) compared to 9.6% (10/104) among heterosexuals. Proportions of RHI among MSM were highest at SHCs (28.9%), followed by GPs (15.4%), other/unknown test locations (16.7%), and hospitals (6.8%, *p* < 0.001). Proportions of RHI among heterosexuals were highest at GPs (16.2%, *p* = 0.39). For MSM and heterosexuals, there was no significant difference in RHI proportions between people of Western and non-Western origin (Table 2).

### Factors Associated With Recency

Univariable and multivariable analyses were performed to identify factors associated with RHI among MSM (Table 2). Univariable analyses showed lower odds of being diagnosed with a RHI among MSM diagnosed at hospitals (OR: 0.18, 95% CI: 0.06–0.51), GPs (0.45, 0.23–0.88), or other/unknown test locations (0.50, 0.11–2.35) compared to MSM diagnosed at SHCs. MSM of older age, of Western origin, residing outside Amsterdam, and who acquired their infection in the Netherlands had slightly lower ORs for RHI, but not significantly. In the multivariable model, only test location remained significantly associated with diagnosis of RHI in MSM.

### Comparison With Epidemiological Data

We compared proportions of RHI based on the RITA algorithm to proportions of RHI based on three epidemiological measures as described in the methods section: (1) prior negative HIV test result ≤6 months before diagnosis, (2) CD4 count ≥500 cells/mm^3^ (10) combined with AIDS-defining illnesses and viral load (<400 copies/ml) at HIV diagnosis, and (3) 1 & 2 combined. Of 692 people included in our study, 446 (64%) had a reported previous negative HIV test result in the ATHENA cohort. Of those, 104 (23.3%) had a negative test result in the past 6 months (Table 3). A relatively high degree of misclassification was observed if recent HIV test results (<6 months before HIV diagnosis) were compared to RITA results. In total, 58 patients with an available RITA test result had a recent negative HIV-test prior to HIV-diagnosis. Of those 23/58 (39.7%) were correctly classified as RHI, but 35/58 (60.3%) misclassified as established HIV-infection with RITA. After correction for ‘false’ established cases, the proportion of patients with RHI would rise from 19.2 to 25.1%.

**Table 3:** Recent HIV infections based on routinely collected epidemiological data at HIV diagnosis (last negative test result and CD4 count) vs. RITA algorithm results.

	**RHI based on RITA algorithm**				
	**RHI**	**Established infection**	**Not RITA tested**	**Total**	**%RHI (unknown excluded)**
**RHI based on recent negative HIV test**					
RHI: ≤6 months before diagnosis	23	35	46	104	23.3
>6 months before diagnosis	41	190	111	342	
Unknown	13	145	88	246	
**RHI based on CD4 count at diagnosis, FRR corrected** ^**1**^					
RHI: CD4 >500 cells/mm^3^	47	111	69	227	34.7
CD4 ≤500 cells/mm^3^	29	248	150	427	
Unknown	1	11	26	38	
**2, false established cases corrected with 1**					
RHI	58	125	89	272	41.3
Established infection	18	237	131	386	
Unknown	1	8	25	34	
**Total**	77	370	245	692	

1 *False Recent Rate (FRR) corrected taking into account AIDS-defining illness and viral load (<400 copies/ml) at HIV diagnosis*.

*^2^Note in this comparison that a person with a negative test result >6 months before diagnosis may be still be recently infected. RITA, recent infection testing algorithm; RHI, recent HIV infection*.

Furthermore, proportions of RHI based on CD4 count (≥500 cells/mm^3^), a rough indicator for recency (18), were much higher (34.7%) compared to proportions of RHI based on recent HIV negative test (23.3%) or the RITA algorithm (17.2%). Correcting false established cases based on CD4 count ≥500 cells/mm^3^ and a recent HIV negative test, resulted in a higher concordance with the RITA algorithm (*n* = 58), but also in more patients identified as RHI (41.3%).

## Discussions

The proportions of recent HIV infections (RHI) based on the algorithm for biomarker-assays (RITA) were 20.4% among MSM and 9.6% among heterosexuals. SHC showed highest proportions of RHI (27%), followed by GPs (15%), hospitals (5%), and other/unknown locations (11%). In a multivariable model among MSM, test location was the only factor significantly associated with RHI. A comparison between RITA outcomes and recency based on routinely collected epidemiological data showed discrepancies that suggest an underestimation of RHI with the biomarker algorithm.

This is the first study to examine RHI at various test locations in the Netherlands including SHCs, GPs and hospitals. The comprehensive data from the ATHENA cohort enabled us to follow the RITA algorithm for RHI (17) and to compare with other indicators for recency based on epidemiological information, such as last negative HIV test results and CD4 counts.

Our results on RHI illustrate that populations tested for HIV at SHCs, GPs, and hospitals are different. HIV testing at GPs and hospitals is more often associated with HIV indicator conditions, while SHCs target high risk populations and offer repeat testing (21). MSM in the Netherlands are advised to test for HIV at least every 6 months (22), resulting in a higher proportion of RHI among MSM at SHCs compared to heterosexuals and patients tested at other locations. GPs contribute substantially to testing and diagnoses of STIs (21), but to lesser extent for HIV (23, 24). STI guidelines for GPs recommend to routinely test MSM and persons with multiple sex partners for HIV (25), but previous studies showed that low-threshold HIV testing among high-risk populations is insufficient at GPs (23, 24). Information on sexual identity is not registered, and both GP and patients may be reluctant to discuss sexual risks at consultations (23, 26). However, incorporating sexual preference in patient records may contribute to broader test offers by GPs and earlier detection of HIV among MSM. HIV indicator based testing is included in GP guidelines and can reduce barriers to obtain sexual histories. At hospitals, proportions of RHI are lowest (8%), and obviously more associated with HIV indicator conditions (16, 23), but also in this setting improvement of guideline uptake may be warranted (27–29).

Comparison with international data on RHI infections is hampered by the use of different RHI assays and window phase variability, next to differences in study populations. However, proportions of RHI among MSM and heterosexuals were comparable to those of England, Northern Ireland and Wales, who used a similar RITA algorithm with an avidity test (AxSYM EIA) (2, 10, 30). They reported proportions of RHI between 22 and 27% among MSM and 8–9% among heterosexuals in 2009–2011 and 2015. In France, a different assay (EIA-RI) was used in 2003–2008 with a proportion of RHI of 25% among newly diagnosed individuals. The false recency rate was not assessed with the ECDC algorithm but estimated from two cohorts of patients (31). The proportion of RHI in Ireland was considerably lower, 14% among MSM and 8% among heterosexuals, but a shorter window phase was described (4–6 months), with a high proportion of diagnoses abroad (8). Proportions of RHI were higher in European countries that used the BED-immunoassay, with proportions of 35% among MSM and 21% among heterosexuals in Germany (2008–2014) (9), 45 and 21% in Sweden (2003–2010) (12), and 31 and 15% in Spain (2006-2008) (11).

High proportions of RHI among MSM are associated with higher testing frequency and ongoing transmission. Repeat testing among MSM has increased in the Netherlands over the years (21), but there was no increasing trend in the proportion of RHI. Likely, this is masked by a decline in HIV transmission over the years in the MSM population or the 3 year study period could be too short to show an increase. We also observed that the proportion of RHI decreased with increasing age, which is consistently reported by other European countries (9–12, 31). Nonetheless, 15% of newly diagnosed MSM aged 45 years or older in our study was recently infected, which indicates ongoing HIV transmission in this age group. Although risk behavior can be similar among older and younger adults, older adults may perceive themselves at less risk for acquiring HIV and report less use of preventive measures including condoms (32, 33). Prevention to reduce risk behavior and to enhance test uptake may also have an impact on MSM of older age. Furthermore, efforts are needed to effectively increase HIV testing rates among heterosexuals. HIV testing in the Netherlands concentrates on heterosexuals originating from high endemic countries and more than half of the heterosexuals included had a non-Western background. RHI proportions were low among both Western and non-Western individuals.

The Architect avidity has been described as an assay with a relative high sensitivity and specificity compared to some other incidence assays; it correctly classified recent HIV in 70% of cases and correctly classified established HIV infection in 95% of cases in a validation study by Hassan et al. (34). We compared the performance of RITA with routinely collected data of last HIV negative test results. It showed that the RITA classification is not that robust and frequent mismatches were found when the results were compared with data on negative HIV test results in the 6 months prior to diagnosis. As testing history was not available for everyone, we also compared the RITA classification with a more complete variable but less specific indicator for RHI: CD4 count ≥500 cells/mm^3^; which is considered the lower limit of the normal range in uninfected individuals. A French study reported similar proportions of RHI (36%) based on CD4 count ≥500 cells/mm^3^ (18). It should be noted that CD4 counts vary widely between individuals during disease progression, and may drop to <500 cells/mm^3^ during acute infections and can be ≥500 cells/mm^3^ up to several years after diagnosis (35), explaining the higher proportion of RHI compared to the RITA classification. As the RITA algorithm and alternative measurements lack precision, the use of multi-assay algorithms (MAA) has been suggested, as it significantly improves the prediction of HIV infection recency (34). The validation study of Hassan et al. (34) showed with regression analyses that the combination of the Architect avidity and the INNO-LIA assay has the potential to identify a higher proportion of recent HIV infections as, individually, the assays have a predicted risk of being recent of 77 and 79%, respectively but in combination the risk increases to 98% (34). However, more research seems needed on costs and feasibility of these combined approaches.

There are some other study limitations to report. First, only patients from the Amsterdam region were included. Although 22% of all cases in the Netherlands are diagnosed in this region (16), results may not be generalisable to other regions in the Netherlands. HIV testing might be more common or accepted in Amsterdam compared to other regions, possibly resulting in higher rates of frequent testing and earlier detection of HIV. Second, for only 65% of newly diagnosed patients stored samples were available for avidity testing with a decreasing number of samples available over time, especially for MSM and Western individuals. Possibly, an earlier start of ART treatment in 2015 among Western individuals (27% in 2013 to 53% in 2015 within a month after diagnosis) compared to non-Western individuals (32–33%) could have resulted in a smaller availability of pre-ART samples. As other characteristics were similar, we expect selection bias to be limited.

In conclusion, although the use of the avidity assay for surveillance purposes may help targeting prevention programs, its robustness is limited and its added value, especially for the MSM population, may decline over time with improved, repeat HIV testing and data collection on testing history. Furthermore, sample collection and logistics would be labor intensive and costly when implemented in all 24 Dutch HIV treatment centers. Therefore, routine incorporation of the avidity assay and the RITA algorithm as part of the national surveillance at all HIV treatment centers in the Netherlands seem neither feasible nor useful in its current form. Instead of the avidity assay, taking its limitations into account, future estimations of RHI could be based on the date of last HIV negative test or in combination with CD4 count and Western Blot patterns (for acute infections) and may act as an alternative measure for HIV recency.

## Data Availability Statement

The datasets presented in this article are not readily available because Statistical information or data for own research purposes can be requested by submitting a research proposal (https://www.hiv-monitoring.nl/english/research/research-projects/). For correspondence: hiv.monitoring@amc.uva.nl. Requests to access the datasets should be directed to hiv.monitoring@amc.uva.nl.

## Ethics Statement

Ethical review and approval was not required for the study on human participants in accordance with the local legislation and institutional requirements. Written informed consent for participation was not required for this study in accordance with the national legislation and the institutional requirements.

## Amsterdam RITA Study Group

Kees Brinkman (OLVG), Arne van Eeden (DC klinieken), Suzanne Jurriaans (Amsterdam UMC-AMC), Jan Prins (Amsterdam UMC-AMC), Martijn van Rooijen (Public Health Service Amsterdam), Dominique Verhagen (MC Jan van Goyen), Marjolein Heitmüller (Amsterdam UMC-VUmc), Edgar J.G. Peters (Amsterdam UMC, location VUmc), Paul Smits (former Slotervaart), Saskia Vrouenraets (former Slotervaart), Peter Reiss (SHM).

## Author Contributions

IS and EO designed the study. IS and FB analyzed the data. IS, FB, and EO drafted the manuscript. AS, AD, TL, GB, and BB contributed to the interpretation of the results and critically revising the manuscript for important intellectual content, and all authors approved the manuscript for publication. All authors contributed to the article and approved the submitted version.

## Conflict of Interest

The authors declare that the research was conducted in the absence of any commercial or financial relationships that could be construed as a potential conflict of interest.
