# Supplementary material for: Monitoring Recently Acquired HIV Infections in Amsterdam, The Netherlands: The Attribution of Test Locations
Source: Front Reprod Health. 2021 Feb 10;3:568611. doi: 10.3389/frph.2021.568611 (PMC9580630; doi:10.3389/frph.2021.568611)
Supplement: Supplementary file 1 [file Table_1.DOCX]

| **Supplemental table.** Proportions of recent HIV infections by year, mode of transmission and test location, region of Amsterdam, 2013-2015 | | | | |
| --- | --- | --- | --- | --- |
|  | **Recent infection** | | |  |
|  | **2013** | **2014** | **2015** |  |
|  | **% (n/N)** | **% (n/N)** | **% (n/N)** | **p-value** |
| **Mode of transmission** | |  |  |  |
| MSM | 21.3 (27/127) | 16.9 (20/118) | 24.4 (19/78) | 0.72 |
| Heterosexual male | 15.0 (3/20) | 4.8 (1/21) | 7.1 (1/14) | 0.39 |
| Heterosexual female | 6.7 (1/15) | 0.0 (0/13) | 19.0 (4/21) | 0.19 |
| Other/unknown | 0.0 (0/5) | 0.0 (0/6) | 11.1 (1/9) | 0.33 |
| **Test location** |  |  |  |  |
| Sexual health centre | 24.2 (16/64) | 22.5 (16/71) | 42.1 (16/38) | 0.09 |
| General practice | 22.2 (12/54) | 6.7 (3/45) | 16.1 (5/31) | 0.60 |
| Hospital | 7.5 (3/40) | 2.9 (1/34) | 4.9 (2/41) | 0.29 |
| Other/unknown | 0.0 (0/7) | 12.5 (1/8) | 16.7 (2/12) | 0.29 |
| ***Test location by mode of transmission*** | |  |  |  |
| **Sexual health centre** |  |  |  |  |
| MSM | 26.2 (16/61) | 24.2 (16/64) | 42.4 (14/33) | 0.16 |
| Heterosexual | 0.0 (0/5) | 0.0 (0/4) | 33.3 (1/3) | 0.14 |
| Other/unknown | 0.0 (0/0) | 0.0 (0/1) | 50.0 (1/2) | 0.48 |
| **General practice** |  |  |  |  |
| MSM | 20.0 (8/40) | 9.4 (3/32) | 15.6 (3/19) | 0.51 |
| Heterosexual | 28.6 (4/44) | 0.0 (0/11) | 16.7 (2/12) | 0.38 |
| Other/unknown | 0.0 (0/0) | 0.0 (0/2) | 0.0 (0/0) | - |
| **Hospital** |  |  |  |  |
| MSM | 13.6 (3/22) | 0.0 (0/16) | 4.5 (1/22) | 0.23 |
| Heterosexual | 0.0 (0/14) | 6.7 (1/15) | 8.3 (1/12) | 0.32 |
| Other/unknown | 0.0 (0/4) | 0.0 (0/3) | 0.0 (0/7) | - |
| **Other/unknown** | |  |  |  |
| MSM | 0.0 (0/4) | 25.0 (1/4) | 25.0 (1/4) | 0.36 |
| Heterosexual | 0.0 (0/2) | 0.0 (0/4) | 12.5 (1/8) | 0.43 |
| Other/unknown | 0.0 (0/1) | 0.0 (0/0) | 0.0 (0/0) | - |
